# Supplementary material for: Development of a computer-aided design software for the quantitative evaluation of aesthetic damage
Source: PLoS One. 2019 Dec 18;14(12):e0226322. doi: 10.1371/journal.pone.0226322 (PMC6919621; doi:10.1371/journal.pone.0226322)
Supplement: S1 Code site — 1. (ZIP) [file pone.0226322.s001.zip › [EESC_jr][Projeto]Relatorio_07_21/reports/templates_zip/gentelella-master.zip!/gentelella-master/vendors/DateJS/reports/lcov-report/core/time_span.js.html]

Code coverage report for core/time\_span.js


# Code coverage report for core/time\_span.js

## Statements: 20.65% (19 / 92)      Branches: 1.72% (1 / 58)      Functions: 16% (4 / 25)      Lines: 20.65% (19 / 92)      Ignored: none

All files » core/ » time\_span.js

```
|  |  |  |
| --- | --- | --- |
| 1 2 3 4 5 6 7 8 9 10 11 12 13 14 15 16 17 18 19 20 21 22 23 24 25 26 27 28 29 30 31 32 33 34 35 36 37 38 39 40 41 42 43 44 45 46 47 48 49 50 51 52 53 54 55 56 57 58 59 60 61 62 63 64 65 66 67 68 69 70 71 72 73 74 75 76 77 78 79 80 81 82 83 84 85 86 87 88 89 90 91 92 93 94 95 96 97 98 99 100 101 102 103 104 105 106 107 108 109 110 111 112 113 114 115 116 117 118 119 120 121 122 123 124 125 126 127 128 129 130 131 132 133 134 135 136 137 138 139 140 141 142 143 144 145 146 147 148 149 150 151 152 153 154 155 156 157 158 159 160 161 162 163 164 165 166 167 168 169 170 171 172 173 174 175 176 | 1   1 5         1 5         1 1 1 5 5 5 5               1                                                                                                                                                                                                                                                     1 1                         1       1   1   1 | ``` (function () { 	"use strict"; 	var gFn = function (attr) { 		return function () { 			return this[attr]; 		}; 	}; 	 	var sFn = function (attr) { 		return function (val) { 			this[attr] = val; 			return this; 		}; 	}; 	var attrs = ["years", "months", "days", "hours", "minutes", "seconds", "milliseconds"]; 	var addSetFuncs = function (context, attrs) { 		for (var i = 0; i < attrs.length ; i++) { 			var $a = attrs[i], $b = $a.slice(0, 1).toUpperCase() + $a.slice(1); 			context.prototype[$a] = 0; 			context.prototype["get" + $b] = gFn($a); 			context.prototype["set" + $b] = sFn($a); 		} 	}; 	/** 	 * new TimeSpan(milliseconds); 	 * new TimeSpan(days, hours, minutes, seconds); 	 * new TimeSpan(days, hours, minutes, seconds, milliseconds); 	 */ 	var TimeSpan = function (days, hours, minutes, seconds, milliseconds) { 		if (arguments.length === 1 && typeof days === "number") { 			var orient = (days < 0) ? -1 : +1; 			var millsLeft = Math.abs(days); 			this.setDays(Math.floor(millsLeft / 86400000) * orient); 			millsLeft = millsLeft % 86400000; 			this.setHours(Math.floor(millsLeft / 3600000) * orient); 			millsLeft = millsLeft % 3600000; 			this.setMinutes(Math.floor(millsLeft / 60000) * orient); 			millsLeft = millsLeft % 60000; 			this.setSeconds(Math.floor(millsLeft / 1000) * orient); 			millsLeft = millsLeft % 1000; 			this.setMilliseconds(millsLeft * orient); 		} else { 			this.set(days, hours, minutes, seconds, milliseconds); 		}   		this.getTotalMilliseconds = function () { 			return	(this.getDays() * 86400000) + 					(this.getHours() * 3600000) + 					(this.getMinutes() * 60000) + 					(this.getSeconds() * 1000); 		}; 		 		this.compareTo = function (time) { 			var t1 = new Date(1970, 1, 1, this.getHours(), this.getMinutes(), this.getSeconds()), t2; 			if (time === null) { 				t2 = new Date(1970, 1, 1, 0, 0, 0); 			} 			else { 				t2 = new Date(1970, 1, 1, time.getHours(), time.getMinutes(), time.getSeconds()); 			} 			return (t1 < t2) ? -1 : (t1 > t2) ? 1 : 0; 		};   		this.equals = function (time) { 			return (this.compareTo(time) === 0); 		};   		this.add = function (time) { 			return (time === null) ? this : this.addSeconds(time.getTotalMilliseconds() / 1000); 		};   		this.subtract = function (time) { 			return (time === null) ? this : this.addSeconds(-time.getTotalMilliseconds() / 1000); 		};   		this.addDays = function (n) { 			return new TimeSpan(this.getTotalMilliseconds() + (n * 86400000)); 		};   		this.addHours = function (n) { 			return new TimeSpan(this.getTotalMilliseconds() + (n * 3600000)); 		};   		this.addMinutes = function (n) { 			return new TimeSpan(this.getTotalMilliseconds() + (n * 60000)); 		};   		this.addSeconds = function (n) { 			return new TimeSpan(this.getTotalMilliseconds() + (n * 1000)); 		};   		this.addMilliseconds = function (n) { 			return new TimeSpan(this.getTotalMilliseconds() + n); 		};   		this.get12HourHour = function () { 			return (this.getHours() > 12) ? this.getHours() - 12 : (this.getHours() === 0) ? 12 : this.getHours(); 		};   		this.getDesignator = function () { 			return (this.getHours() < 12) ? Date.CultureInfo.amDesignator : Date.CultureInfo.pmDesignator; 		};   		this.toString = function (format) { 			this._toString = function () { 				if (this.getDays() !== null && this.getDays() > 0) { 					return this.getDays() + "." + this.getHours() + ":" + this.p(this.getMinutes()) + ":" + this.p(this.getSeconds()); 				} else { 					return this.getHours() + ":" + this.p(this.getMinutes()) + ":" + this.p(this.getSeconds()); 				} 			}; 			 			this.p = function (s) { 				return (s.toString().length < 2) ? "0" + s : s; 			}; 			 			var me = this; 			 			return format ? format.replace(/dd?\|HH?\|hh?\|mm?\|ss?\|tt?/g, 			function (format) { 				switch (format) { 				case "d": 					return me.getDays(); 				case "dd": 					return me.p(me.getDays()); 				case "H": 					return me.getHours(); 				case "HH": 					return me.p(me.getHours()); 				case "h": 					return me.get12HourHour(); 				case "hh": 					return me.p(me.get12HourHour()); 				case "m": 					return me.getMinutes(); 				case "mm": 					return me.p(me.getMinutes()); 				case "s": 					return me.getSeconds(); 				case "ss": 					return me.p(me.getSeconds()); 				case "t": 					return ((me.getHours() < 12) ? Date.CultureInfo.amDesignator : Date.CultureInfo.pmDesignator).substring(0, 1); 				case "tt": 					return (me.getHours() < 12) ? Date.CultureInfo.amDesignator : Date.CultureInfo.pmDesignator; 				} 			} 			) : this._toString(); 		}; 		return this; 	}; 	addSetFuncs(TimeSpan, attrs.slice(2)); 	TimeSpan.prototype.set = function (days, hours, minutes, seconds, milliseconds){ 		this.setDays(days \|\| this.getDays()); 		this.setHours(hours \|\| this.getHours()); 		this.setMinutes(minutes \|\| this.getMinutes()); 		this.setSeconds(seconds \|\| this.getSeconds()); 		this.setMilliseconds(milliseconds \|\| this.getMilliseconds()); 	};     	/** 	 * Gets the time of day for this date instances.  	 * @return {TimeSpan} TimeSpan 	 */ 	Date.prototype.getTimeOfDay = function () { 		return new TimeSpan(0, this.getHours(), this.getMinutes(), this.getSeconds(), this.getMilliseconds()); 	};   	Date.TimeSpan = TimeSpan;   	Eif (typeof window !== "undefined" ) { 		// keeping API compatible for v1.x  		window.TimeSpan = TimeSpan; 	} }()); ``` |
```

Generated by istanbul at Sun Nov 16 2014 12:08:33 GMT-0800 (PST)
